# Predicted trends in long-term breast cancer survival in England and Wales

**DOI:** 10.1038/sj.bjc.6603668

**Published:** 2007-04-03

**Authors:** L M Woods, B Rachet, N Cooper, M P Coleman

**Affiliations:** 1 Cancer Research UK Cancer Survival Group, Non-Communicable Disease Epidemiology Unit, Department of Epidemiology and Population Health, London School of Hygiene & Tropical Medicine, Keppel Street, London WC1E 7HT, UK; 2 National Cancer Intelligence Centre, Health and Social Care Division, Office for National Statistics, 1 Drummond Gate, London SW1V 2QQ, UK

**Keywords:** breast cancer, women, relative survival, long-term, trends, England and Wales

## Abstract

Trends in long-term relative survival from breast cancer are examined for women diagnosed in England and Wales up to 2001, using both period and hybrid approaches. Large improvements in long-term survival are predicted. Women with breast cancer still experience persistent excess mortality up to at least 20 years after diagnosis.

Compared with other common malignancies, breast cancer in women has a relatively good prognosis in the short and medium term (Berrino *et al*, 2003; Coleman *et al*, 2004). Where long-term survival has been examined, it has mostly been shown that women with breast cancer continue to experience excess mortality into the second and third decades after their diagnosis (Duncan and Kerr, 1976; Langlands *et al*, 1979; Brinkley and Haybittle, 1984; Adami *et al*, 1986; Zahl and Tretli, 1997; Joensuu *et al*, 1999). This excess mortality may be due to the existence of ‘micro-metastases’ – disseminated cancer cells present even in women with apparently localised disease at diagnosis (Slade *et al*, 2005). Because previous survival studies have been based on cohort or complete approaches, trends in long-term survival have involved women diagnosed from the 1940s up to the 1970s, at least 10 years before the important advances in diagnosis and management that occurred during the 1980s.

We have used statistical methods designed to reflect the recent gains in short-term survival, to provide the most ‘up-to-date’ predictions of long-term survival possible, namely for women diagnosed in England and Wales during the period 1971–2001 and followed up to 2003.

## MATERIALS AND METHODS

**Figure 1:**
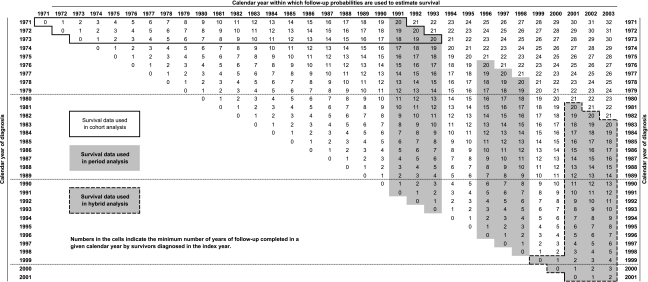
Illustration of the survival times used in cohort, period and hybrid approaches to survival estimation.

Data were obtained from the Office for National Statistics on all women diagnosed in England and Wales with invasive breast cancer in 1971–2001 and followed up until 31 December 2003. To these, we applied the inclusion and exclusion criteria recently reported (Coleman *et al*, 2004), before estimating relative survival up to 20 years after diagnosis. Relative survival estimates the disease-specific survival by subtracting the background general population mortality from the overall mortality observed among the cancer patients. It is the most defensible method of examining long-term disease survival, because it does not rely upon accurate reporting of cause of death (Ederer *et al*, 1961). We used both period and hybrid approaches to estimate relative survival and they are contrasted with the classical cohort approach in Figure 1.

The period approach (Brenner and Gefeller, 1997) derives the most up-to-date estimates of survival by incorporating the probabilities of death derived from the most recent year or period for which follow-up data are available, while excluding survival probabilities derived from earlier periods (left censoring). The calculation of period survival is analogous to that of life expectancy at birth from a period life table. It was used to produce sets of estimates of long-term survival for women with breast cancer who were alive at some point either during the period 1991–1993 or during 1996–1998. These period survival estimates are best interpreted as the predicted probability of survival for a patient diagnosed within those periods.

Hybrid analysis is a modification of the period approach that combines both cohort and period techniques (Brenner and Rachet, 2004). It is preferable in scenarios where information on the patients' vital status becomes available more rapidly than the reporting of incident cases, because it enables unbiased estimates of survival; it was applied for the period 1999–2003. Cohort survival estimates from the first and second years of follow-up for women diagnosed during 1999–2001 were combined with period estimates of relative survival for the third to twentieth years of follow-up observed among women alive at any point during the interval 2001–2003 (Figure 1).

Relative survival rates were all estimated with a maximum likelihood approach for individual tumour data (Estève *et al*, 1990) using the algorithm *strel* (Cancer Research UK Cancer Survival Group, 2006) implemented in STATA™ (StataCorp, 2006). Background mortality was taken from period- and deprivation-specific national life tables in every analysis (Cancer Research UK Cancer Survival Group, 2004). Survival estimates were derived for all women aged 15–99 years at diagnosis, and separately for the three broad age groups 15–49, 50–69 and 70–99 years. Changes in survival over a 10-year period were derived from a variance-weighted linear regression of survival estimates for the periods 1991–1993, 1996–1998 and 1999–2003. Overall survival estimates for all women aged 15–99 years at diagnosis were age-standardised using the population structure of women diagnosed with breast cancer in England and Wales during the period 1986–1990, to enable comparison with published data (Coleman *et al*, 1999, 2004).

## RESULTS

Overall predicted long-term survival has improved markedly since 1991–1993, with a 17–20% improvement in predicted 10- and 20-year relative survival over the 10-year interval from 1991–1993 to 1999–2003 (Table 1). The predicted improvement in long-term survival was better for women aged 50 and over at diagnosis (18–24%), than for those aged 15–49 years (13–14%).

Overall 10-year relative survival, for all women diagnosed with invasive breast cancer in England and Wales between 1999 and 2003 in the age range 15–99 years, is predicted to exceed 72%. More than 64% of recently diagnosed women are predicted to survive to the 20th anniversary of their diagnosis.

**Figure 2:**
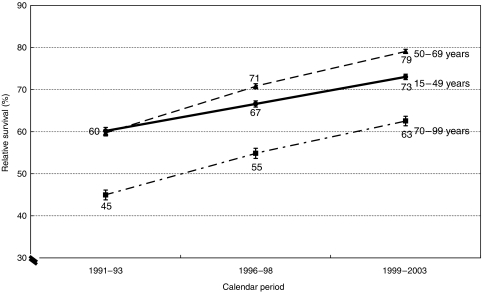
Predicted trends up to 2003 in 10-year relative survival (%) from breast cancer, by age at diagnosis, with 95% confidence intervals: women, England and Wales.

In 1991–1993, women diagnosed at ages 15–49 years had higher predicted 10-, 15- and 20-year survival than older women, but a decade later, their predicted survival was lower than for women diagnosed aged 50–69 years (Figure 2). By contrast, 1-year survival rates and trends were very similar in these age groups (data not shown). Older women (70–99 years) experienced the lowest survival, particularly 10 years after diagnosis (63%). However, although the difference between 10- and 20-year survival was similar for all three age groups among women diagnosed in 1991–1993 (about 10%), the difference was less marked among older women diagnosed since 1999. This reflects the larger recent increase in long-term survival among women aged 50 and over than among those aged 15–49 years.

## DISCUSSION

This study predicts substantial increases in long-term survival from invasive breast cancer among women diagnosed over the period 1991–2003, particularly for those aged 50 years or more. It also shows persisting excess mortality up to at least 20 years after diagnosis.

We have previously documented large and continuing improvements in survival in England and Wales over this period using conventional approaches (Coleman *et al*, 1999, 2004). This study extends those reports by providing predictions of long-term survival for women diagnosed very recently, as well as demonstrating how this predicted survival has changed over time.

The rising trend in long-term survival in breast cancer in England and Wales probably reflects the combined impact of several important changes in its diagnosis and management over the last two decades. These include the widespread adoption of adjuvant chemotherapy, as recommended by the 1985 world overview (Baum and Houghton, 1999), the increasing use of hormonal-based treatments in England and Wales, particularly for post-menopausal women, the use of radiotherapy after breast-conserving surgery (Early Breast Cancer Trialists' Collaborative Group, 2005) and the establishment of the National Health Service Breast Screening Programme for women aged 50–64 years in 1988 (Quinn *et al*, 1995).

In this period-based approach, the survival improvements described reflect the impact of very recent changes in breast cancer management upon short-term survival, combined with the impact of less recent changes upon longer-term survival. Consequently, although the survival rates reported here are more ‘up-to-date’ than conventional estimates, even period estimates cannot yet reflect the full potential impact of recent advances in the diagnosis and treatment of breast cancer on long-term survival, because women contributing to the survival estimates for the 15th to 20th years of follow-up were diagnosed before those advances were made (Figure 1). A further 5–10 years of incidence and follow-up data will enable the impact of these changes in diagnosis and management upon very long-term survival to be formally assessed.

Comparing the 10-year survival rates with those estimated for 15–20 years after diagnosis shows that relative survival for breast cancer continues to decrease throughout the two decades following diagnosis. This is consistent both with early work, and with a more recent analysis of women diagnosed with breast cancer in Finland up to 1999 (Brenner and Hakulinen, 2004). Our data further suggest that this tendency applies to women diagnosed as recently as 1981 and that it has not diminished over time, although it is not yet possible to assess the impact of the changes implemented during the late 1980s upon survival beyond 15 years after diagnosis.

Our study highlights the much better prognosis for women diagnosed in the first few years of the twenty-first century than for women diagnosed during the early 1990s. However, the continued existence of late mortality from breast cancer suggests that the treatments given for breast cancer, at least up to the mid-to-late 1980s, have not yet eliminated the continuing risk of death due to the disease more than 10 years after initial diagnosis.

## Figures and Tables

**Table 1:** Trends in long-term relative survival (%) predicted for women diagnosed with invasive breast cancer during 1991–1993, 1996–1998 and 1999–2003, by age at diagnosis and time since diagnosis: England and Wales

	**Calendar period of follow-up used in the analyses**									
	**1991–1993 (period approach)**			**1996–1998 (period approach)**			**1999–2003 (hybrid approach)**			
**Age at diagnosis**	**Women^1^**	**Deaths**	**Relative survival (95% CI)**	**Women^1^**	**Deaths**	**Relative survival (95% CI)**	**Women^1^**	**Deaths**	**Relative survival (95% CI)**	**10-year change^2^ in relative survival (95% CI)**
Time since diagnosis										
All ages (15–99), age-standardised										
10 years	256 954	48 423	54.3 (53.8–54.9)	314 026	49 216	63.7 (63.2–64.3)	329 498	62 038	71.5 (71.0–72.0)	17.1 (16.4–17.9)
15 years			47.8 (46.9–48.7)			56.4 (55.5–57.3)			67.3 (66.4–68.1)	19.5 (18.2–20.8)
20 years			44.4 (43.1–45.7)			52.0 (50.8–53.3)			64.3 (62.9–65.7)	19.7 (17.9–21.6)
										
*15–49 years*										
10 years	67 553	7 451	60.2 (59.4–61.0)	82 750	7 665	66.6 (65.9–67.3)	84 871	8 842	73.0 (72.4–73.7)	12.8 (11.8–13.9)
15 years			54.0 (53.1–54.9)			60.2 (59.3–61.0)			67.6 (66.8–68.3)	13.7 (12.5–14.8)
20 years			49.8 (48.8–50.8)			55.8 (54.8–56.7)			63.4 (62.5–64.2)	13.7 (12.3–15.0)
										
*50–69 years*										
10 years	125 214	19 236	59.7 (59.0–60.4)	154 006	18 524	70.8 (70.2–71.4)	165 459	21 445	79.1 (78.6–79.6)	19.1 (18.3–20.0)
15 years			51.9 (51.1–52.7)			62.7 (61.9–63.5)			75.2 (74.6–75.9)	23.5 (22.4–24.5)
20 years			48.0 (47.0–49.0)			57.6 (56.6–58.6)			71.7 (70.7–72.6)	23.8 (22.3–25.2)
										
*70–99 years*										
10 years	64 187	21 736	45.0 (43.8–46.1)	77 270	23 027	54.9 (53.6–56.1)	79 168	31 751	62.6 (61.4–63.7)	17.6 (16.0–19.2)
15 years			39.1 (37.4–40.8)			46.7 (44.9–48.5)			59.0 (57.1–60.8)	19.7 (17.2–22.3)
20 years			37.4 (33.9–41.0)			43.2 (40.1–46.3)			58.7 (55.2–62.0)	21.4 (16.5–26.3)

1 Number of women alive at some point during the period of follow-up used in the analyses.

2 10-year change in relative survival (absolute fitted increase, %) derived from a variance-weighted linear regression of relative survival estimates for the periods 1991–1993, 1996–1998 and 1999–2003.
